# Evaluating Secrecy Capacity for In-Body Wireless Channels

**DOI:** 10.3390/e21090858

**Published:** 2019-09-03

**Authors:** Muhammad Faheem Awan, Xiao Fang, Mehrab Ramzan, Niels Neumann, Qiong Wang, Dirk Plettemeier, Kimmo Kansanen

**Affiliations:** 1 Department of Electronic Systems, Norwegian University of Science and Technology, NTNU, NO-7491 Trondheim, Norway; 2 Chair of RF and Photonics Engineering, Technische Universität Dresden, 01067 Dresden, Germany (X.F.) (M.R.) (N.N.) (Q.W.) (D.P.)

**Keywords:** implanted medical devices, wireless leadless cardiac pacemaker, in-body wireless channels, security and privacy, physical layer security, secrecy capacity

## Abstract

The next generation of implanted medical devices is expected to be wireless, bringing along new security threats. Thus, it is critical to secure the communication between legitimate nodes inside the body from a possible eavesdropper. This work assesses the feasibility of securing next generation multi-nodal leadless cardiac pacemakers using physical layer security methods. The secure communication rate without leakage of information to an eavesdropper, referred to as secrecy capacity, depends on the signal-to-noise ratios (SNRs) of the eavesdropper and legitimate channels and will be used as a performance metric. Numerical electromagnetic simulations are utilized to compute the wireless channel models for the respective links. These channel models can be approximated with a log-normal distribution which can be used to evaluate the probability of positive secrecy capacity and the outage probability of this secrecy capacity. The channels are modeled for three different frequency bands and a comparison between their secrecy capacities is provided with respect to the eavesdropper distance. It has been found that the positive secrecy capacity is achievable within the personal space of the human body for all the frequency bands, with the medical implant communication systems (MICS) band outperforming others.

## 1. Introduction

The technological advancements in implanted medical devices have resulted in the rapid growth of personal health systems which include popular wireless medical devices like cardiac pacemakers, glucose monitors, and implantable cardioverter defibrillators (ICDs). These wireless medical devices are less invasive than traditional wired solutions and provide proper diagnosis and treatment.

One of the most important medical device is the cardiac pacemaker, which helps to maintain cardiac rhythms. There are almost one million pacemaker implantations worldwide annually [1]. The current generation of these pacemakers consists of a subcutaneous implant connected to electrodes in the right atrium and right ventricle by leads, whereas the next generation is expected to be wireless in all aspects including connectivity between the subcutaneous implant and electrodes. The electrodes of the so-called leadless cardiac pacemakers in the heart chambers will be wirelessly synchronized with each other and also with the subcutaneous implant which will be used to configure the leadless pacemakers and that acts as a relay for external devices.

Besides the unquestionable benefits of leadless pacemakers such as less invasive surgery, also some disadvantages arise. One of the key issues is to protect the life saving device from intruders and eavesdroppers. Successful eavesdropping results in fetching of patient’s confidential information (medical/non-medical) or executing different types of attacks (e.g., forging and data manipulation). Moreover, it may facilitate the modification of implant configuration without knowledge of the patient or physician [2]. Thus, the wireless nature of these devices could be a safety risk and must be secured from threats like eavesdropping, data tampering and device modification. This work does not cover all aspects related to the security of implanted medical devices and focuses only on secrecy capacity for in-body channels with the assumption of a passive eavesdropper outside the body. The passive eavesdropper only intercepts the communication without any active attacks. This can lead to future active attacks like data tampering, man-in-the-middle attack and un-authorized access. For example, in the case of SNR estimation spoofing, the active eavesdropper can adapt the strategy of reporting a worse SNR than the legitimate receiver, albeit having the better SNR, reflecting the secrecy capacity to be positive. Therefore, if the transmitter unit is not able to distinguish that forgery attempt, then eavesdropper will end up partially decoding the confidential information. On the other way around, the eavesdropper can report its SNR to be better than legitimate channel, which results in utilizing the resources from the transmitter unit [3]. Moreover, jamming also directly affects the secrecy metrics, because it changes the estimated and actual legitimate receiver SNR. A detailed investigation of active attacks is beyond the scope of this work.

Halperin et al.’s [4] work is believed to be the pioneer study in investigating the security risks of implanted medical devices and proofs with off the shelf antennas and external programmers (An external device used for data collection and configuration of a cardiac pacemaker) that the wireless nature of these devices can be exploited to control them. This work was followed by numerous studies providing the methodologies and techniques to secure wireless implanted medical devices [5,6,7,8]. Most of the work is focused on methods based on computational cryptography. Another alternative could be utilizing the physical layer to provide secure communication via information theoretic measures. In addition, risk management and evaluation are part of international standards for implanted medical devices [9,10]. Therefore, physical layer security (PLS) assessments together with traditional cryptographic measures should be part of these standards.

The theory of information theoretic security was initially suggested by Shannon [11] in 1949. Wyner [12] extended Shannon’s work in 1975 and introduced the secrecy capacity of the Gaussian wiretap channel. Secrecy capacity is a communication rate with which the legitimate nodes can communicate securely in the presence of an eavesdropper. Secrecy capacity can be achieved if the legitimate channel signal-to-noise ratio (SNR) is better than the eavesdropper’s (Eve’s) channel. There have been considerable efforts to secure wireless networks [13] based on the PLS methods. Jameel et al. in [14] provided a comprehensive survey on cooperative relaying and jamming strategies for PLS methods whereas [15] proposed the multicasting cooperation strategy to enhance the security in large networks. Also, Neshenko et al. [16], provided an extensive survey on different types of vulnerabilities. However, these methods mainly focus on free space wireless networks and cannot be directly applied to in-body scenarios because of completely different media.

Information theoretic measures or security via PLS depend on wireless channel characteristics for securing the communication between legitimate nodes. These characteristics may involve received signal strength, angle of arrival, phase or the inherent noise in the wireless channels that degrades the signal-to-noise ratio [17,18,19,20]. Therefore, channel modeling is the key aspect to evaluating the possibilities of using the PLS methods for securing the information content between legitimate nodes.

Channel characterizations subject to the human body are commonly carried out in electromagnetic (EM) computational simulation tools like computer simulation technology (CST) [21] or High frequency structure simulator (HFSS) [22], phantom or in-vivo experiments. IEEE 802.15.6 is the specified standard for wireless body area network (WBAN) in which Medical Implant Communication (MICS) band is specified as the communication standard for the implant to implant communication. The MICS frequency band covers the frequency range of 402–405 MHz. Literature is also available on channel modeling in other frequency bands such as Wireless Medical Telemetry Service (WMTS, 608–614 MHz), ISM 868 MHz, Ultra Wide Band (3.1–5.1 GHz), and ISM 2.4 GHz [23,24,25]. Similarly, Kadel et al. [26], provide comparisons between different channel models proposed in the literature for on-body to on-body scenarios in WBAN for 900 MHz and 3.1–10 GHz. Their simulation results are derived from a 2-D human model without considering the impacts of different human organs. Therefore, the results can be considered as less precise. Moreover, their channel models cannot be utilized for our application scenario, because the transmitter and receiver are considered on the body surface whereas in our case the transmitter is located inside the heart and the receiver is positioned in the subcutaneous space.

This work evaluates the secrecy capacity for in-body channels and explores the PLS methods for privacy and security of multi-nodal leadless cardiac pacemaker (LCP). The in-body to in-body legitimate channel and in-body to off-body eavesdropper channels are simulated in a computer simulation tool to derive the path loss models for channel attenuation. In comparison to channel models presented in [26], we evaluated the application-based channel model that involves propagation through organs in the cardiac scenario. In addition, the evaluation of channel models is based on a 3-D anatomical human model with an EM simulation method which is precise and considers all the electric properties of human organs and propagation characteristics of electromagnetic waves inside the human body. Based on the cardiac application scenarios, three different channel models, in-body to in-body, in-body to subcutaneous and in-body to off-body, are developed and lower frequency bands are mainly considered, i.e., MICS, WMTS and ISM. The channel attenuation is utilized to evaluate the respective link signal-to-noise ratio (SNR) for determining the probability of positive secrecy capacity along with the outage probability of the secrecy capacity. The secrecy capacity analysis is carried out in all the frequency bands under investigation. It has been observed that the MICS band outperforms other bands in terms of achieving secrecy capacity in near vicinity of the human body. However, if the practical considerations of antenna dimensions are to be considered, then the ISM 868 MHz is the viable choice.

The rest of the paper is organized as follows. Section 2 provides the system model and methodology and Section 3 contains the results. The discussions are provided in Section 4 and conclusions in Section 5.

## 2. System Model and Methodology

**Figure 1:**
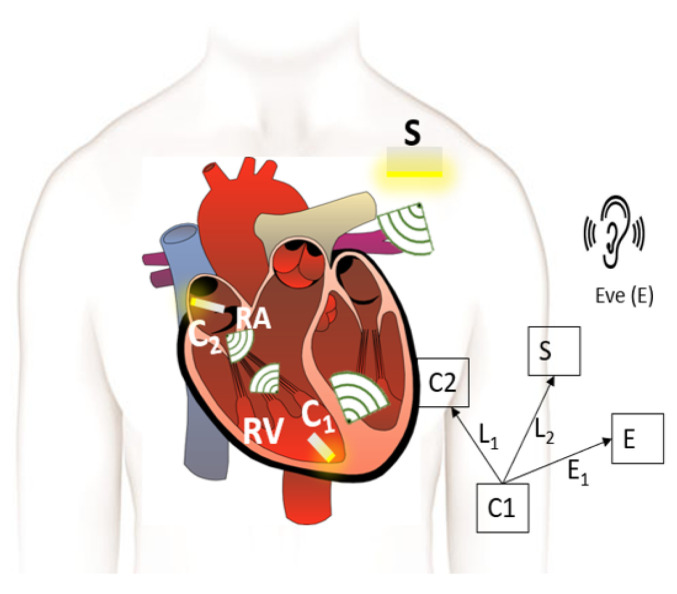
Multi-nodal leadless cardiac pacemaker scenario with leadless capsules C1 and C2, subcutaneous implant S and an eavesdropper E.

This section provides the system model for a multi-nodal leadless cardiac pacemaker which consists of a leadless pacemaker in the right ventricle and right atrium of the human heart, a subcutaneous implant in the pectoral pocket under the shoulder and an eavesdropper outside the body as shown in Figure 1. The leadless pacemakers, also referred to as capsules, communicate wirelessly with each other and with the subcutaneous implant. We define three links, two legitimate and one eavesdropper link—the link between *C1* and *C2* referred to as *L1*, the link between *C1* and the subcutaneous implant (*L2*) and the link between *C1* and the eavesdropper (*E1*).

The legitimate nodes use MICS, WMTS, and ISM 868 MHz frequency bands in order to communicate with each other. To evaluate the secrecy capacity, the channels for all communication links between legitimate nodes and with the eavesdropper are modeled in CST. First, the methodology to evaluate the secrecy capacity and its dependence on channel models is provided, followed by channel modeling using electromagnetic simulations.

### 2.1. Methodology

The wireless in-body network shown in Figure 1 depicts *C1* communicating with *C2* and *S*, whereas the eavesdropper (Eve) in the near vicinity of the body is attempting to spy on the communication. The legitimate nodes can communicate securely by using the secure transmission rate which is the maximum achievable confidential communication rate without the disclosure of information to Eve. All the noise sources are considered to be white and Gaussian e.g., thermal noise, shot noise of the Rx and Tx and we do not expect any nonlinearities from Rx, Tx and the communication medium. Therefore, by using [27] for an additive Gaussian wiretap channel, the instantaneous secrecy capacity is expressed as

(1) $$\begin{array}{c} \begin{array}{c} C_{s}=C_{r}-C_{e} \end{array} \end{array}$$

where $C_{r}$ and $C_{e}$ are the channel capacities of legitimate and eavesdropper link respectively, which can be expressed as

(2) $$\begin{array}{c} C_{r}=\frac{1}{2}\log_{2}\left(1+\gamma_{r}\right) \end{array}$$

(3) $$\begin{array}{c} C_{e}=\frac{1}{2}\log_{2}\left(1+\gamma_{e}\right) \end{array}$$

Consequently, Equation (1) can be followed from Equation (2) and Equation (3) as

(4) $$C_{s}=\left\{\begin{array}{cc} \frac{1}{2}\log_{2}\left(1+\gamma_{r}\right)-\frac{1}{2}\log_{2}\left(1+\gamma_{e}\right), & \mathrm{if}\;\gamma_{r}>\gamma_{e}.\\ 0, & \mathrm{otherwise}\;[27]. \end{array}\right.$$

$\gamma_{r}$ represents the legitimate channel SNR and $\gamma_{e}$ shows the SNR of Eve’s channel. Equation (4) expresses that $C_{s}$ is positive when the legitimate channel SNR is greater than Eve’s channel i.e., ($\gamma_{r}>\gamma_{e}$). With positive $C_{s}$, the legitimate nodes can communicate securely. Furthermore, the SNR of a link can be computed as

(5) $$\begin{array}{c} \begin{array}{c} \gamma_{i}=\frac{P*|h_{i}{|}^{2}}{W_{i}},\;i\;\in \;\left(r,e\right) \end{array} \end{array}$$

where $|h_{e}{|}^{2}$, $|h_{r}{|}^{2}$ represents the channel attenuations of the associated links, *P* is the transmitted power which is set to −16 dBm (power restrictions on implanted devices [28]) and *W* is the constant noise power. Therefore, channel attenuations are the only source of variations in channel capacities. Thus, in order to compute the secrecy capacity for in-body implanted legitimate nodes, the channel model for attenuation between the legitimate nodes and eavesdropper must be analyzed.

### 2.2. Channel Modeling

The simulation is performed in the anatomical human model provided by the CST family of voxel models. The transmitting antenna is an electrically small antenna which has a far-field radiation similar to a Hertzian dipole. Hence, for simplicity, it is represented by a Hertzian dipole source in the simulation. The ideal Hertzian dipole source does not take the mismatch and structural loss of the real antenna into consideration, however, in the practical case, these losses cannot be ignored, which will increase the path loss and deteriorate the channel SNR. Three polarizations of the Hertzian dipole have been investigated. In order to detect the electric and magnetic fields at different distances from the transmitting dipole, ideal electric and magnetic probes are utilized. Based on the LCP scenario, the transmitting antenna is placed at the vertex of the right ventricle which is the actual pacemaker placement site.

**Figure 2:**
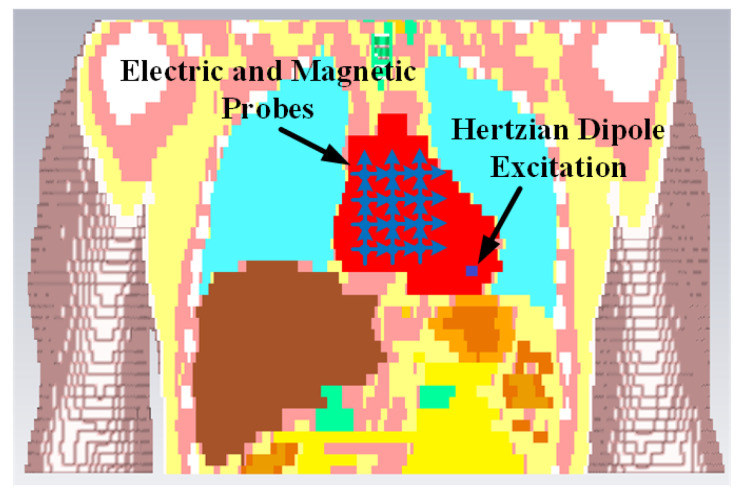
Cross section view of simulation scenario of Intra-cardiac to Intra-cardiac channel models.

**Figure 3:**
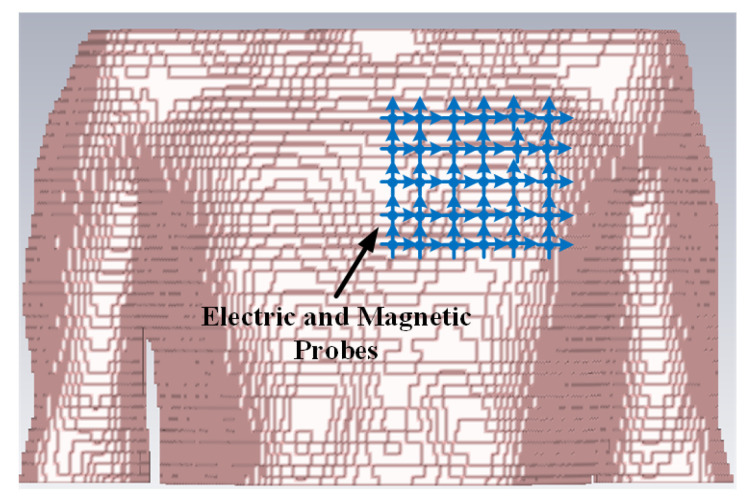
Cross section view of simulation scenario of Intra-cardiac to Subcutaneous channel models.

**Figure 4:**
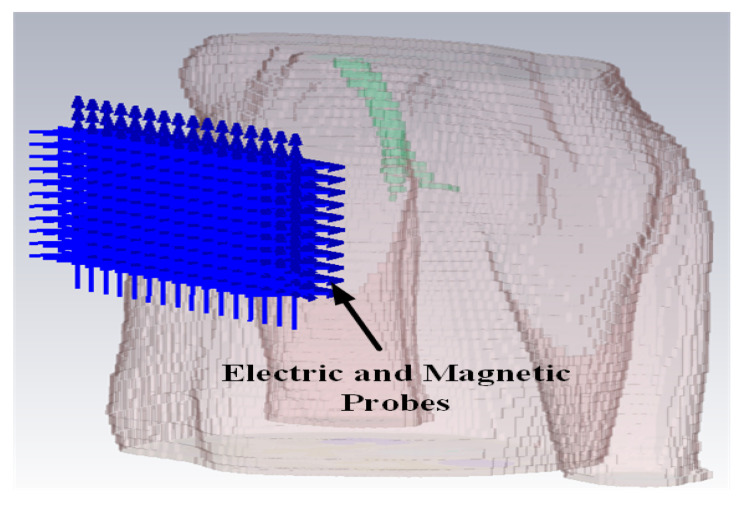
Side view of the simulation scenario of Intra-cardiac to Off-Body channel models.

**Figure 5:**
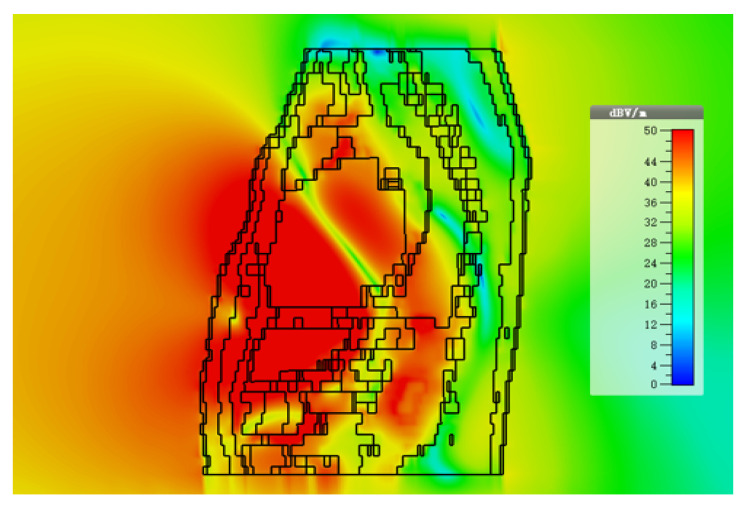
Radiation of EM waves outside the body.

The intra-cardiac simulation scenario or an intra-cardiac link (*L1*) is shown in Figure 2, where a pacemaker is placed in the right ventricle and different receiving probes in the right atrium. The intra-cardiac to the subcutaneous channel or link between *C1* and *S* (i.e., *L2*) is illustrated in Figure 3. The probes are placed 2 cm below the skin surface under the left collar bone of the human body and is regarded as the actual placement site for the subcutaneous device. Figure 4 shows Eve’s channel (*E1*) where the probes are positioned a few centimeters away from the body surface in front of the chest. The probes are located at a site with maximum received power. This is considered as the worst case scenario for the pacemaker (best case scenario for Eve) under practical conditions, i.e., limited antenna size for Eve. Figure 5 shows the spatial distribution of the strength of EM radiation outside the body, with maximum received power in front of the body showing the best case scenario for Eve. The power distribution at each position is evaluated using the Poynting vector which can be expressed as

(6) $$S_{\left(x,y,z\right)}\left(t\right)=E_{\left(x,y,z\right)}\left(t\right)\times H_{\left(x,y,z\right)}\left(t\right)$$

where $E_{\left(x,y,z\right)}\left(t\right)$ and $H_{\left(x,y,z\right)}\left(t\right)$ are the time-domain electric and magnetic field vector. The average received power at a single position is determined from all three *x*,*y*,*z* polarized electric and magnetic probes. Finally, the path loss is calculated from the ratio of the averaged received power at the observation point to the average transmitted power. For computing simplicity of secrecy capacity and outage probability, a single path loss model is often desirable for both in-body and off-body links. Under the practical limitation of Eve using an antenna with limited size, this is an acceptable simplification. However, when exploring the absolute limits of the received power, the different nature of the loss inside and outside the body should be considered. The power lost inside the body is dissipated to heat (lossy medium) whereas the path loss outside the body is because of the spatial distribution of the electromagnetic wave. Using a theoretical antenna/receiver system that is able to receive all power radiated from the body, this path loss may be compensated completely. Due to the fact that this kind of system is extremely difficult to implement, especially without disclosure of Eve’s intends, the practical case of limited antenna size and path loss outside the body will be used for the calculations. Therefore, the derivation of the path loss model is based on the following equation

(7) $$PL_{dB}=PL_{0,dB}+10n\log\left(\frac{d}{d_{0}}\right)+N\left(0,\sigma_{dB}\right)$$

where $PL_{0,dB}$ is the path loss at the reference distance $d_{0}$ and *n* is the path loss exponent. The cumulative distribution function (CDF) can be approximated by a log-normally distributed random variable N(0,σ) with zero mean and standard deviation σ.

**Figure 6:**
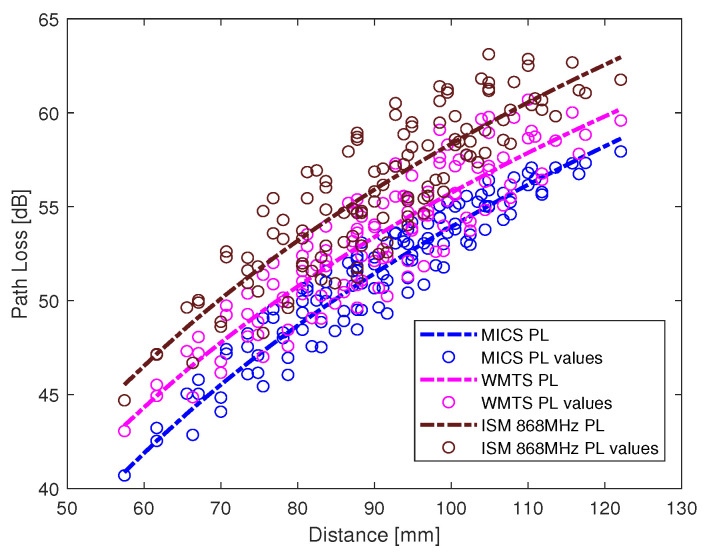
Intra-Cardiac link (*L1*) pathloss models.

**Figure 7:**
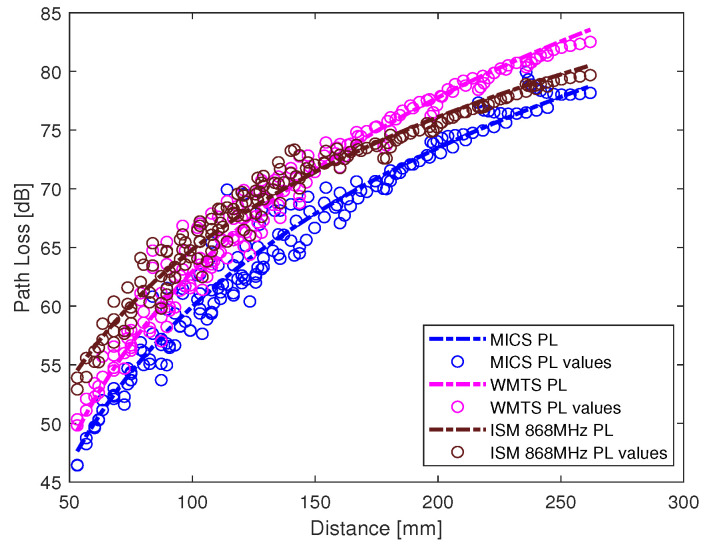
Off body link (*E1*) path loss models.

**Figure 8:**
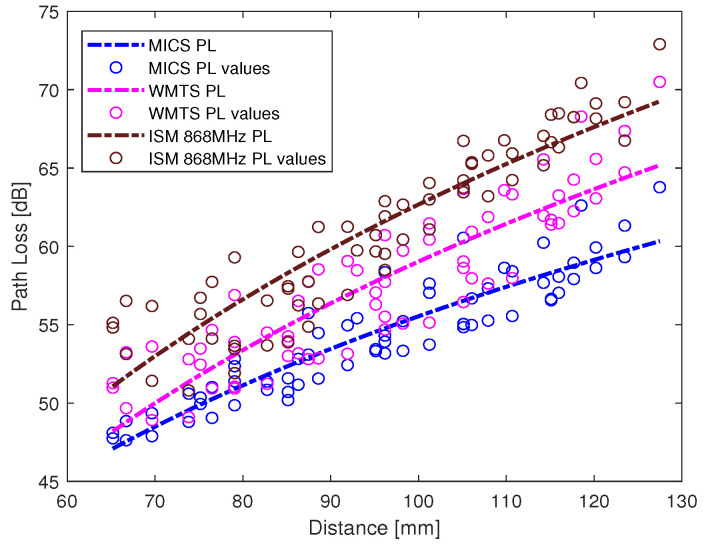
Subcutaneous link (*L2*) path loss models

The path loss values along with the fitted model for intra-cardiac simulation or *L1* link is shown in Figure 6. It shows that the path loss varies with respect to the frequency band used. The intra-cardiac to off-body channel models (*E1*) consist of free space and complex human body tissue medium. As mentioned before, for the computational simplicity of secrecy capacity and outage probability, a single path loss model is often desirable. Therefore, a single path loss model curve with average path loss exponent is extracted as shown in Figure 7. The slope of the fitted curves clearly indicates that when the receiver is in the near vicinity of the human body, the lossless medium influence can be neglected and as the receiver is being moved away there is slightly decreasing change in the slope indicating the influence of the presence of the free space medium. At distance larger than 150 mm, the path loss at ISM 868 MHz becomes less than that in the WMTS band. The complex nature of human organs causes reflection and scattering which may cause increased received power at certain locations outside the human body due to constructive interference. This effect is more prominent at higher frequencies (i.e., smaller wavelengths). The intra-cardiac to subcutaneous channel model or *L2* link is shown in Figure 8. The increasing tendency of path loss is similar to that of intra-cardiac path loss. Table 1 shows the summary of all the nine models depicted using EM simulations for the corresponding legitimate and eavesdropper links in the frequency bands under investigation.

## 3. Results

In this section, the secrecy capacity analysis of a multi-nodal leadless cardiac pacemaker is provided. As enlisted in Table 1, all the path loss models for channel attenuation are modeled with a log-normal distribution. Thus, the corresponding SNR values $\gamma_{r}$ and $\gamma_{e}$ will also follow the log-normal distribution at any measuring point with mean and standard deviation ($\mu_{r}$,$\sigma_{r}$) and ($\mu_{e}$,$\sigma_{e}$), respectively. The fundamental parameters in the context of secrecy capacity are the probability of positive secrecy capacity ($P_{pc_{s}}$) and the outage probability of secrecy capacity ($OP_{c_{s}}$). The secrecy capacity is positive when Eve’s link SNR is inferior to legitimate link’s SNR and is referred to as positive secrecy capacity. The outage probability of secrecy capacity can be defined by setting a fixed secrecy rate ($R_{s}$) and can be computed with respect to the eavesdropper distance. As $\gamma_{r}$ and $\gamma_{e}$ are mutually independent and log-normally distributed, then for a single realization of a legitimate channel and eavesdropper channel, the probability of positive secrecy capacity can be expressed as

(8) $$\begin{array}{c} \begin{array}{cc} P(C_{s}>0) & =P(\gamma_{r}>\gamma_{e}) \end{array} \end{array}$$

Similarly, by setting a fixed secrecy rate ($R_{s}$), the outage probability of secrecy capacity can be expressed as

(9) $$\begin{array}{c} \begin{array}{cc} P(C_{s}<R_{s}) & =1-P(C_{s}>R_{s}) \end{array} \end{array}$$

After simplification (as provided in detail in Appendix A and adapted from [29]), $P_{pc_{s}}$ can be represented in the form of Q-function for log-normal channels [19,29] as

(10) $$\begin{array}{c} \begin{array}{c} P\left(C_{s}>0\right)=1-Q\left(\frac{\ln\mu_{\gamma_{e}}-\ln\mu_{\gamma_{r}}+8\left(b^{2}-a^{2}\right)}{4\sqrt{a^{2}+b^{2}}}\right) \end{array} \end{array}$$

whereas the outage probability ($OP_{c_{s}}$) can be expressed as

(11) $$\begin{array}{c} \begin{array}{c} P\left(C_{s}<Rs\right)=Q\left(\frac{\ln\frac{\mu_{\gamma_{r}}}{\mu_{\gamma_{e}}}+8\left(b^{2}-a^{2}\right)-Rs\ln2}{4\sqrt{a^{2}+b^{2}}}\right) \end{array} \end{array}$$

where $\mu_{\gamma_{e}}$ and $\mu_{\gamma_{r}}$ represents the mean SNR of respective links (legitimate and Eve Link, as expressed in Equation (5)). In addition, $a=\frac{\sigma_{r}ln10}{40}$ and $b=\frac{\sigma_{e}ln10}{40}$, where *a* and *b* is the standard deviation of the Gaussian distribution which corresponds to a log-normal distribution (if $\sigma_{e}$ is the standard deviation of $\gamma_{e}$, then $a=\frac{\sigma_{e}ln10}{40}$). As shown in Figure 1 and numbers enlisted in Table 1, there are two legitimate links, one between node *C1* and *C2* i.e., *L1* whereas other between *C1* and *S* i.e., *L2*—and the eavesdropper link (*E1*). Thus, a separate analysis is provided for both the legitimate links considering the same link for Eve.

### 3.1. Probability of Positive Secrecy Capacity ($P_{pc_{s}}$)

**Figure 9:**
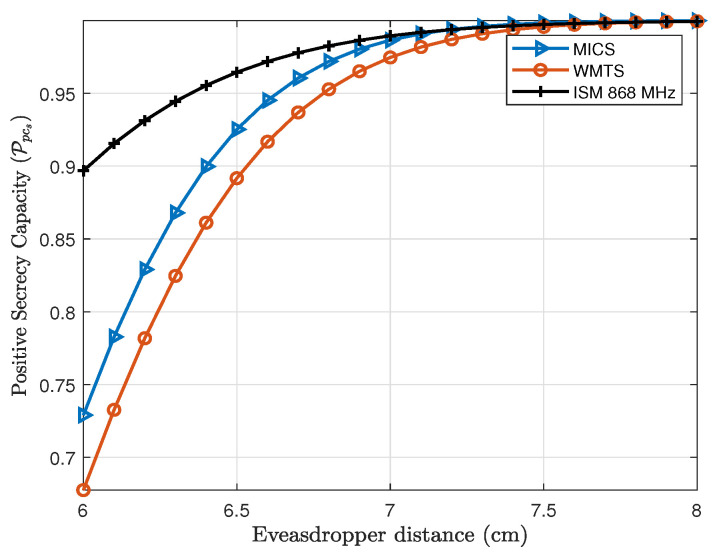
Probability of positive secrecy capacity of intra-cardiac (C1 and C2 or L1) link for the frequency bands under investigation.

**Figure 10:**
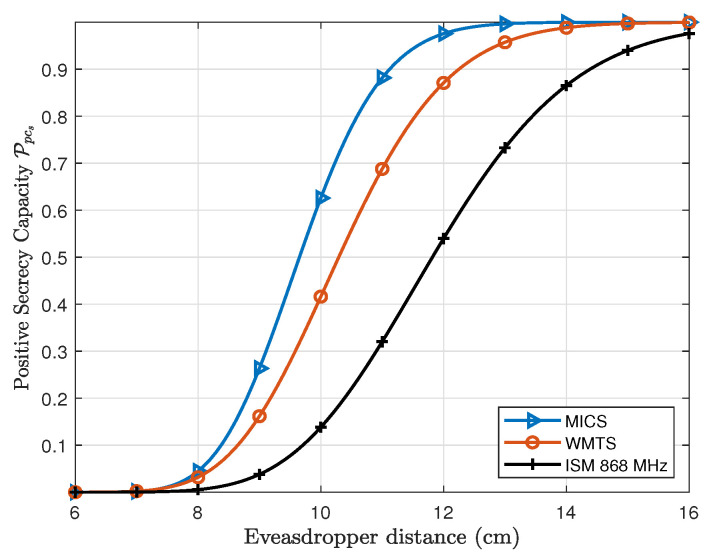
Probability of positive secrecy capacity of Subcutaneous link (L2) for frequency bands under investigation.

First, the intra-cardiac link (*L1*) and the eavesdropper link (*E1*) are considered. Thus, by using Equation (10), the $P_{pc_{s}}$ for the intra-cardiac link is shown in Figure 9, whereas the $P_{pc_{s}}$ for the subcutaneous link (*C1* and *S* or *L2*) is shown in Figure 10. In case of the intra-cardiac link, a fixed distance of 8cm between node *C1* and *C2* is considered and the eavesdropper distance is varied. By considering Eve at a distance of 6 cm which ultimately means being attached directly to the body of a patient over the heart on the chest, $P_{pc_{s}}$ is approximately 68% for WMTS, around 75% for MICS and about 90% for ISM 868 MHz. When the eavesdropper is moved away just about 2 cm, the positive secrecy capacity approaches to approx. 100% for all the frequency bands. Normally, in case of lower frequencies with small in-body distance between nodes the probability of secrecy capacity is high. As shown in Figure 10, ISM 868 MHz has higher $P_{pc_{s}}$ than MICS and WMTS, but this is because of the small path loss exponent “*n*” and also the smaller standard deviation of path loss than MICS and WMTS. However, after 7.6cm MICS and WMTS have higher secrecy capacity than ISM 868 MHz.

Similarly, in case of the subcutaneous link (*L2*), the distance between *C1* and *S* is fixed to 12 cm which is the average distance between the capsule in the right ventricle and subcutaneous implant. If the eavesdropper is considered to be attached to the human body over the heart, the $P_{pc_{s}}$ is about 0%. However, if an eavesdropper moves away from the body $P_{pc_{s}}$ increases and approaches to approx. 100% at a distance of 15 cm in case of MICS and WMTS band, whereas about 99.97% at a distance of 20 cm for ISM 868 MHz. In case of subcutaneous link (*L2*) as expected the MICS band has higher $P_{pc_{s}}$ at close premises to the body than WMTS and ISM 868 MHz.

### 3.2. Outage Probability of Secrecy Capacity ($OP_{c_{s}}$)

**Figure 11:**
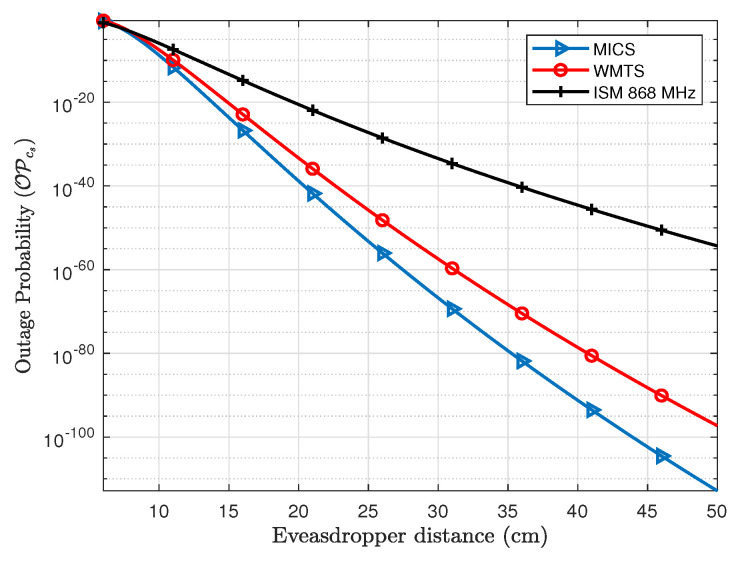
Comparison of outage probability of secrecy capacity for intra-Cardiac link for the investigated frequency bands.

**Figure 12:**
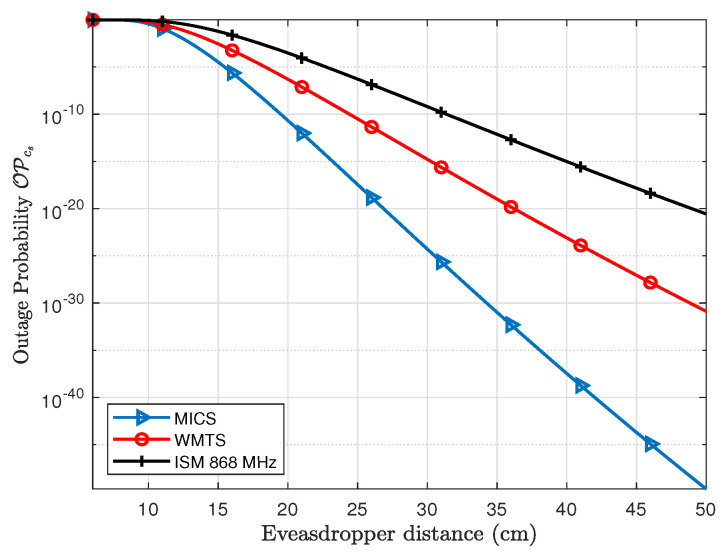
Comparison of Outage probability of secrecy capacity for subcutaneous link for the investigated frequency bands.

In order to evaluate the outage probability of secrecy capacity, a secure communication rate between legitimate nodes is needed to be established. In case of pacemakers, the communication rate required to transmit different physiological parameters varies, e.g., the required communication rate for electrocardiography (ECG) is around 2.5–250 kbps, whereas electromyography (EMG) requires around 650 kbps [30]. If the secrecy rate is set to about 1 bps/Hz which for a bandwidth of 1 MHz is equivalent to 1 Mbps, the outage probability of secrecy capacity can be provided by using Equation (11). Figure 11 shows the outage probability of fixed secrecy rate for the legitimate link (*L1*). The distance of link *L1* is fixed to 8 cm and the eavesdropper distance is varied. The ${\mathrm{OP}}_{c_{s}}$ of link *L1* at an Eve distance of 6 cm is about 84% for MICS, 81% for WMTS and 50% for ISM 868 MHz and approaches nearly to 2% at Eve’s distance of about 8 cm. Similarly for subcutaneous link (*L2*), the ${\mathrm{OP}}_{c_{s}}$ is shown in Figure 12. In case of the subcutaneous link, the distance between *C1* and *S* is about 12 cm. The ${\mathrm{OP}}_{c_{s}}$ at an Eve distance of 12 cm is about 28% for MICS, 48% for WMTS, and 86% for ISM 868 MHz whereas the ${\mathrm{OP}}_{c_{s}}$ approaches to approximately 0.076%, 3.1% and 21%, respectively, at a distance of Eve of 16 cm in case of MICS, 18 cm in case of WMTS and 22 cm in case of ISM 868 MHz. However, for a decent level of safety, lower outage probabilities are desirable.

## 4. Discussion

So far, a scenario with an eavesdropper outside the body with a small antenna has been analyzed where multinodal leadless pacemakers are implanted in the right atrium and right ventricle of the human heart, along with a subcutaneous implant beneath the shoulder under the skin. Our findings show that the physical layer security methods with the use of the secrecy capacity is viable and can be an efficient alternative to secure the implanted medical devices on a physical layer. This is because the human body is a lossy medium for electromagnetic propagation, inherently providing high channel attenuation to off-body links e.g., the eavesdropper link *E1*. Eve being outside the body has an advantage of compensating the high path loss by use of different types of antennas and thus can improve the quality of a link with high gain antennas. Higher gain antennas can have a reception from a greater distance with high SNR, thus reducing the secrecy capacity rate. However, these kind of outperforming antennas are realized at a cost of larger dimensions of the antenna. The dimension of an antenna with provided gain can be estimated using

(12) $$A_{e}=\frac{c^{2}G}{4\pi f^{2}}$$

where $A_{e}$ is the effective aperture, *G* is the antenna gain, and *f* is the frequency. Thus, in order to analyze the effect of antenna gain at the eavesdropper, the outage probability of secrecy capacity is evaluated by considering Eve having an ideal antenna with 10 dBi gain. As seen in Equation (12), the effective aperture (i.e., size) of antennas increases with its gain. As a result, 10 dBi was chosen to balance antenna dimensions and gain. Table 2 enlists the comparison of outage probabilities with and without antenna gain for both intra-cardiac (*L1*) and subcutaneous link (*L2*). The antenna aperture is given exemplary in quadratic dimensions for better imagination. In addition, a personal space of 50 cm is considered for an individual with a pacemaker. Eve will be noticed when operating within this space. It can be seen that for the intra-cardiac link, the outage probabilities are extremely low even with a high gain antenna on Eve’s side. This is because of the low path loss between intra-cardiac leadless pacemakers. For the subcutaneous link, the MICS band provides the best results with an outage probability of $10^{-6}$ at Eve’s distance of 25 cm and $10^{-26}$ at 50 cm. For an individual personal space of 50 cm, the worst results are for ISM 868 MHz with an antenna gain of 10 dBi at Eve’s side and have the outage probability of secrecy capacity to be $10^{-9}$ at 50 cm. But with required dimensions of e.g., 30 × 30 cm${}^{2}$, it will hardly be possible for an eavesdropper to remain unobserved.

These results prove that there is a good probability to achieve positive secrecy capacity in near premises of the patient and also a secure communication rate can be achieved for the cardiac application, even if the eavesdropper is attached to the patient’s body. The cardiac application rates mainly correspond to heart rate, blood pressure (1.92 kbps), respiratory rate (1 kbps), pulse rate (2.4 kbps) and ECG with maximum required data rate of 2.5–250 kbps [30,31]. These data rates are well below the fixed secure communication rate for which Eve’s distances are specified.

In case of achieving positive secrecy capacity and low outage probability, MICS band could be the best choice to be used for implanted medical devices. However, it is difficult to develop a small efficient antenna with good reflection coefficient taking into consideration its practical realization at lower frequencies. On the other hand, there is a good possibility to develop small efficient antennas at higher frequencies, but the losses definitely increase as well as the secrecy capacity is achieved at a greater distance than in the MICS band. Thus, from practical considerations, ISM 868 MHz will be a good choice for developing small antennas with good efficiency and acceptable outage probabilities of the secrecy capacity.

## 5. Conclusions

The secrecy capacity for wireless in-body channels has been evaluated in different frequency bands that include MICS, WMTS and ISM 868 MHz. With an application for multi-nodal leadless cardiac pacemakers, the probability of positive secrecy capacity and outage probability of fixed secure communication rate has been determined for legitimate links, i.e., intra-cardiac link (link *L1* between leadless pacemakers inside the heart) and subcutaneous link (link *L2* between leadless pacemaker inside the heart and subcutaneous implant). By considering an individual personal space of 50 cm, it has been found that the intra-cardiac link is not critical in terms of outage probability of secure communication rate of 1 bps/Hz, even with an antenna gain of 10 dBi for the eavesdropper. The maximum outage probability of the secrecy capacity for a subcutaneous link (*L2*) is $10^{-9}$, for Eve at a distance of 50 cm having an antenna gain of 10 dBi. This corresponds to one patient in a billion which is a very good number considering the application sensitivity and the eavesdropping scenario.

In the future, the impact of active eavesdroppers will be considered on the secrecy metrics of the system. The channel models will also be developed further and refined using more simulations and experiments. The experiments could involve development of phantoms used for in-body experimentation, which is a chemical solution that replicates the dielectric properties of human organs.

## Figures and Tables

**Table 1:** Summary of the path loss models for intra-cardiac (L1), subcutaneous (L2) and off-body eavesdropper (E1) link.

Parameter	MICS (402–405 MHz)			WMTS (608–614 MHz)			ISM 868 MHz (867–869 MHz)		
	L1	L2	E1	L1	L2	E1	L1	L2	E1
PL(d0) (dB)	42.5	48.5	50	44.9	50	52	47.2	53	56.5
d0 (cm)	6	7	6	6	7	6	6	7	6
n	5.12	4.54	4.465	4.86	5.83	4.970	4.99	6.24	3.773
μ	0	0	0	0	0	0	0	0	0
σ (dB)	1.26	1.64	1.379	1.83	2.64	1.275	2.14	2.24	1.143
d (cm)	5.7–12.2	6–13	5.6–24.4	5.7–12.2	6–13	5.6–24.4	5.7–12.2	6–13	5.6–24.4

**Table 2:** Comparison of outage probability (OP) of secrecy capacity for scenarios with and without antenna gain at the eavesdropper.

Frequency Band	Effective Aperture (cm${}^{2}$)		Intra-Cardiac Link (L1)				Subcutaneous Link (L2)			
			OP at 0 dBi		OP at 10 dBi		OP at 0 dBi		OP at 10 dBi	
	For 0 dBi	For 10 dBi	25 cm	50 cm	25 cm	50 cm	25 cm	50 cm	25 cm	50 cm
MICS	21×21	66×66	$10^{-54}$	$10^{-113}$	$10^{-24}$	$10^{-67}$	$10^{-18}$	$10^{-51}$	$10^{-6}$	$10^{-26}$
WMTS	19×19	61×61	$10^{-46}$	$10^{-98}$	$10^{-23}$	$10^{-61}$	$10^{-11}$	$10^{-32}$	$10^{-4}$	$10^{-18}$
ISM 868 MHz	10×10	30×30	$10^{-28}$	$10^{-56}$	$10^{-12}$	$10^{-30}$	$10^{-8}$	$10^{-22}$	${1.8\times 10}^{-1}$	$10^{-9}$

## Abbreviations

The following abbreviations are used in this manuscript:

C1	Leadless pacemaker in Right Atrium
C2	Leadless pacemaker in Right Ventricle
$C_{s}$	Secrecy Capacity
CST	Computer Simulation Technology
CDF	Cumulative Distributive Function
E1	Link between C1 and Eve
ECG	Electrocardiography
EM	Electromagnetic
EMG	Electromyogram
IB2IB	In-Body to In-Body
IB2OFF	In-Body to Off-Body
ICD	Implanted Cardioverter Defibrillator
ISM	Industrial Scientific and Medical Frequency Band
L1	Link between C1 and C2
L2	Link between C1 and subcutaneous implant (S)
LCP	Leadless Cardiac Pacemaker
MICS	Medical Implant Communication Systems
$OP_{c_{s}}$	Outage probability of secrecy capacity
$P_{pc_{s}}$	Probability of Positive Secrecy Capacity
PL	Path Loss
PLS	Physical-Layer Security
RF	Radio Frequency
$R_{s}$	Fixed Secure Communication Rate
Rx	Receiver
SNR	Signal to Noise Ratio
Tx	Transmitter
UWB	Ultrawide Band
WMTS	Wireless Medical Telemetry Service
WBAN	Wireless Body Area Network
WiBEC	Wireless In-body Environment

## Appendix A

(A1) $$\begin{array}{c} \begin{array}{c} P\left(C_{s}>0\right)=\frac{1}{4a\sqrt{2\pi }}\int_{0}^{\infty }\frac{1}{\gamma_{e}}\times \left(1-Q\left(\frac{1}{4b}\ln\frac{\gamma_{e}}{n}\right)\right)\times \exp\left(\frac{1}{2}{\left(\frac{1}{4a}\ln\left(\frac{\gamma_{e}}{m}\right)\right)}^{2}\right)d_{\gamma_{e}} \end{array} \end{array}$$

Consider,

(A2) $$\begin{array}{c} \begin{array}{c} x=\frac{1}{4b\sqrt{2}}\ln\left(\frac{\gamma_{e}}{n}\right) \end{array} \end{array}$$

Then (A1) becomes

(A3) $$\begin{array}{c} \begin{array}{c} P\left(C_{s}>0\right)=\frac{b}{a\sqrt{\pi }}\left(\alpha -\beta \right) \end{array} \end{array}$$

where,

(A4) $$\begin{array}{c} \begin{array}{cc} \alpha & =\int_{-\infty }^{\infty }exp\left(-{\left(\frac{b}{a}\right)}^{2}{\left(x+\frac{1}{4b\sqrt{2}}\ln\left(\frac{n}{m}\right)\right)}^{2}\right)dx=\frac{a\sqrt{\pi }}{b} \end{array} \end{array}$$

(A5) $$\begin{array}{c} \begin{array}{cc} \beta & =\int_{-\infty }^{\infty }Q\left(x\sqrt{2}\right)exp\left(-{\left(\frac{b}{a}\right)}^{2}{\left(x+\frac{1}{4b\sqrt{2}}\ln\left(\frac{n}{m}\right)\right)}^{2}\right)dx \end{array} \end{array}$$

Using Middleton’s work ([32], p. 1072), β can be expressed as

(A6) $$\begin{array}{c} \begin{array}{cc} \beta & =\frac{a\sqrt{\pi }}{b}Q\left(\frac{ln(n/m)}{4\sqrt{a^{2}+b^{2}}}\right) \end{array} \end{array}$$

which follows,

(A7) $$\begin{array}{c} \begin{array}{c} P\left(C_{s}<Rs\right)=Q\left(\frac{\ln\frac{\mu_{\gamma_{r}}}{\mu_{\gamma_{e}}}+8\left(b^{2}-a^{2}\right)-Rs\ln2}{4\sqrt{a^{2}+b^{2}}}\right) \end{array} \end{array}$$

and

(A8) $$\begin{array}{c} \begin{array}{c} P\left(C_{s}>0\right)=Q\left(\frac{\ln\mu_{\gamma_{e}}-\ln\mu_{\gamma_{r}}+8\left(b^{2}-a^{2}\right)}{4\sqrt{a^{2}+b^{2}}}\right) \end{array} \end{array}$$
